# Frozen Cropland Soil in Northeast China as Source of N_2_O and CO_2_ Emissions

**DOI:** 10.1371/journal.pone.0115761

**Published:** 2014-12-23

**Authors:** Shujie Miao, Yunfa Qiao, Xiaozeng Han, Roberta Brancher Franco, Martin Burger

**Affiliations:** 1 Key Laboratory of Mollisols Agroecology, Northeast Institute of Geography and Agroecology, Chinese Academy of Sciences, Harbin, China; 2 Department of Land, Air and Water Resources, University of California Davis, Davis, California, United States of America; DOE Pacific Northwest National Laboratory, United States of America

## Abstract

Agricultural soils are important sources of atmospheric N_2_O and CO_2_. However, in boreal agro-ecosystems the contribution of the winter season to annual emissions of these gases has rarely been determined. In this study, soil N_2_O and CO_2_ fluxes were measured for 6 years in a corn-soybean-wheat rotation in northeast China to quantify the contribution of wintertime N_2_O and CO_2_ fluxes to annual emissions. The treatments were chemical fertilizer (NPK), chemical fertilizer plus composted pig manure (NPKOM), and control (Cont.). Mean soil N_2_O fluxes among all three treatments in the winter (November–March), when soil temperatures are below −7°C for extended periods, were 0.89–3.01 µg N m^−2 ^h^−1^, and in between the growing season and winter (October and April), when freeze-thaw events occur, 1.73–5.48 µg N m^−2 ^h^−1^. The cumulative N_2_O emissions were on average 0.27–1.39, 0.03–0.08 and 0.03–0.11 kg N_2_O_–_N ha^−1^ during the growing season, October and April, and winter, respectively. The average contributions of winter N_2_O efflux to annual emissions were 6.3–12.1%. In all three seasons, the highest N_2_O emissions occurred in NPKOM, while NPK and Cont. emissions were similar. Cumulative CO_2_ emissions were 2.73–4.94, 0.13–0.20 and 0.07–0.11 Mg CO_2_-C ha^−1^ during growing season, October and April, and winter, respectively. The contribution of winter CO_2_ to total annual emissions was 2.0–2.4%. Our results indicate that in boreal agricultural systems in northeast China, CO_2_ and N_2_O emissions continue throughout the winter.

## Introduction

Agricultural cropland is a significant source of the greenhouse gas nitrous oxide (N_2_O) accounting for about 60% of anthropogenic N_2_O [1]. Thus, understanding the sources and temporal variations of N_2_O flux from cropland, as well as the underlying mechanisms for these emissions, is necessary in order to fully account for all the annual greenhouse gas emissions and devise mitigation strategies. Nitrous oxide emissions in agricultural soils result from nitrification and denitrification processes [2], which are regulated by microbial activity, soil moisture, temperature, mineralizable C and N content [3]–[5]. Soil respiration by heterotrophic microorganisms is a major source of CO_2_ returned to the atmosphere from agricultural soil. Historically, the emissions of N_2_O and CO_2_ in high latitudes during winter have mostly been ignored as they are assumed to be small since soil microbial and root activity below 0°C and in frozen soil conditions is low. There is a lack of information on winter N_2_O emissions in agro-ecosystems in middle and high latitude regions. While numerous intensive studies on terrestrial CO_2_ flux from frozen soil have been conducted (e.g. Shi et al. [6]), much less work has been done to quantify N_2_O emissions in boreal cropland during winter [7], [8].

In recent years, a host of studies has highlighted that non-growing season emissions contribute a significant amount of CO_2_ and N_2_O emitted to the atmosphere [9]–[11] although the sources of these non-growing season emissions have not always been clearly determined. Non-growing season N_2_O and CO_2_ emissions have been shown to be related to climate, soil type, management practice, and fertilization [12]–[16]. In boreal agricultural ecosystems, the duration and depth of snow cover directly affect soil temperature, and hence, N_2_O emissions. Non-growing season N_2_O emissions from agricultural and prairie soils have sometimes been attributed to thaw events [10], [17], [18], but N_2_O emissions from frozen agricultural soils are not well understood [16].

The northeast plain region in China is an important food production area [6], where corn-soybean-wheat is the most common rotation system. These rotations are heavily fertilized with synthetic fertilizer, which is sometimes supplemented with livestock manure, and the effects of these amendments on non-growing season N_2_O emissions have not been determined. The growing season in this region is usually five months (from May to September), and the non-growing season is 7 months long (from October to April). April and October (spring/fall) are periods of transition with fluctuations in temperature and occasional freeze-thaw events [6] whereas the period from November to March (winter) is characterized by sustained sub-zero temperatures. In this Black-soil region, Shi et al. [6] reported contributions of non-growing season soil respiration to annual soil CO_2_ emissions of 15.2%, with a contribution of 7.1% from the period when soils were continuously frozen. Meanwhile, N_2_O emissions during the non-growing season in Northeast China have to-date not been measured.

In the present study, a 6-year field experiment encompassing three different fertilization treatments in a corn-soybean-wheat rotation was conducted to determine i) fluxes of N_2_O and CO_2_ during spring/fall and winter; ii) differences in N_2_O and CO_2_ emissions among fertilization treatments, and iii) the contribution of non-growing season N_2_O and CO_2_ emissions to annual emissions of these gases.

## Materials and Methods

### 1. Site description

The study was conducted for six years (2006–2012) as part of an ongoing fertilization and crop rotation field experiment, which was initiated in 1990 in the center of the black soil region in northeastern China, Hailun State Key Agro-ecological Experiment Station, Hailun County, Heilongjiang Province, China (N47°26′, E126°38′). The mean annual air temperature and precipitation are 1.5°C and 550 mm, respectively. More than 65% of the annual precipitation occurs from June to August. The local climate is a semi-humid temperate continental monsoon climate with long, cold winters (November to March). The winter is dry with snow cover beginning in November and snow-melt occurring in early April. The soil was classified as *Pachic Haploborolls* in the US system [19]. The total C of the soil is 27.9 g C kg^−1^ and the total N 2.2 g N kg^−1^ [20]. The pH at the inception of the experiment was 7.02.

### 2. Soil and agronomic management and experimental design

The 6-year experiment was conducted in a corn-soybean-wheat rotation from 2006–2011 (Table 1). The following treatments have been in place since 1990: Control (Cont.) without any amendments, chemical fertilizer (NPK), and chemical and organic fertilizer (composted pig manure) (NPKOM) (Table 2). The synthetic fertilizers were applied at planting and as supplemental N addition in July. After corn and soybean, tillage to 20 cm depth with a ground-driven rotary tiller was conducted in the fall after harvest. After wheat, tillage as above took place after plowing with a five bottom moldboard (20 cm depth) plow. The composted manure was applied preceding fall tillage, which occurred earlier after wheat (August 15) than after corn or soybean crops (October 15) (Table 1). The manure was evenly spread onto the soil surface by hand and immediately incorporated. The soil in all the treatments was bare during winter. No tillage took place in spring.

**Table 1:** Cropping sequence.

Crop	Year	Planting Date	Tillage Date
Corn	2006	May 8	Oct 15
	2009	May 6	Oct 15
Soybean	2007	May 5	Oct 15
	2010	May 9	Oct 15
Wheat	2008	April 7	Aug 15
	2011	April 10	Aug 15

Crop rotation with planting and tillage dates.

**Table 2:** Annual fertilizer and manure inputs.

Crop	Treatment	Nitrogen	Phosphorus (P_2_O_5_)	Potassium (K_2_O)	Organic matter	Carbon
		(kg ha^−1^)				
Soybean	Control	0	0	0	0	
	NPK	20.25	51.75	30	0	
	NPKOM	20.25+36.0^#^	51.75	30	2250	338
Wheat, Corn	Control	0	0	0	0	
	NPK	112.5*	45	30	0	
	NPKOM	112.5* +36.0^#^	45	30	2250	338

The fertilizer and manure inputs in the three fertility treatments for each of the crops. NPK synthetic fertilizer applied only; NPKOM synthetic fertilizer and manure applied. *62.5 kg N ha^−1^ basal N fertilizer as urea at planting and 50 kg N ha^−1^ as supplemental fertilizer as urea in July. ^#^Amount of total N added in the composted manure.

The experimental design was a randomized complete block design with three replicates per treatment. Each replicate plot was 15 m long and 4.2 m wide.

The long-term experiment was performed in accordance with guidelines specified under Hailun State Key Agro-ecological Experiment Station, and no specific permissions were required for these locations and without endangered or protected species in our study location.

### 3. N_2_O and CO_2_ flux measurement

A static chamber method was used to determine N_2_O and CO_2_ flux according to the methodology reported by Li et al. [21]. Immediately after planting, polyvinyl chloride (PVC) bases were placed between the rows. The PVC bases for N_2_O sampling (69×19×2 cm) were inserted 2 cm deep into the soil to allow root growth underneath the chamber area, whereas the bases for CO_2_ sampling (69×19×25 cm) were placed 25 cm deep into the soil to exclude root growth under the chamber area. The bases were removed before harvest and re-inserted into the soil surface after harvest following fall plough. During flux measurements, the 10 cm tall PVC chambers were set atop the bases by inserting the flange of the chamber into a water channel (growing season) or on sticky sponge strips (non-growing season) at the protruding ends of the bases. The location of the bases was marked with flags inserted at each corner of the bases. When the ground was covered with snow, the chambers were set 2 cm deep into the snow at the marked locations in similar fashion as described by Groffman et al. [22].

Flux measurements were conducted twice a week during the growing season and at intervals of 10 days during the non-growing season from May 2006 to April 2012. Sampling was carried out between 10∶00 am and 11∶00 am, a period representing approximately the daily average soil temperature [6]. During each sampling, chamber air was collected at 0, 10, 20 and 30 min with a syringe. The flux measurements at all 9 chamber locations were completed within a one-hour period. 20-ml gas samples were removed from the chambers by inserting the needle of a gas-tight syringe through a septum installed at the top of the closed chamber, and then the gas samples were immediately transferred to pre-evacuated vials. The samples were at greater than atmospheric pressure during transport to the lab.

Gas chromatography (Shimadzu, GC2010, Japan) was used to measure the N_2_O and CO_2_ concentrations in aliquots of 1.0 ml gas. The GC was equipped with an electron capture detector (ECD) with ^63^N_i_ radioactive source using P/Q column to measure N_2_O. The carrier gas was an argon-methane mixture. A methanizer and flame ionization detector (FID) with a Chromosorb 102 column was used to measure CO_2_ concentrations. The carrier gas was dinitrogen. The GC was calibrated for each batch of samples with analytical grade standard gases of N_2_O (208, 298, 497, and 804 ppb N_2_O) and CO_2_ (371, 797, 1203, and 1998 ppm CO_2_) (Haipu Corp, Beijing, China). The minimum detectable flux on this GC was 9×10^−8^ µg N_2_O_–_N m^2 ^h^−1^. The molar mixing ratios of the samples were converted to mass per volume values using ideal gas relations. Soil gas flux (*SF*) was calculated as reported by Guo et al. [23]:
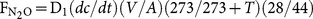
(1)

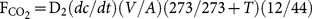
(2)


Where, *F*_N2O_, *F*_CO2_ stand for N_2_O flux in µg N m^−2 ^h^−1^ and for CO_2_ flux in mg C m^−2 ^h^−1^; D_1_, D_2_ for N_2_O and CO_2_ density under the standard conditions, respectively; *dc/dt* for temporal increase in N_2_O and CO_2_ concentration in the chamber headspace determined by linear regression; *V* for effective headspace volume of the chamber (0.0168 m^3^); *A* for the soil area covered by the chamber (0.14 m^2^) and *T* for air temperature (**°**K) inside the chamber. The flux results were accepted if the coefficient of determination (r^2^) of the linear regression for at least three of the four time points was >0.90. Overall, <5% of all the data were discarded because they did not conform to these criteria.

### 4. Annual and seasonal emissions of N_2_O and CO_2_

In addition to calculating average hourly fluxes of N_2_O and CO_2_ for each replicate and season, annual cumulative N_2_O and CO_2_ emissions per season (growing, spring-fall, winter) for each replicate were calculated by assuming that hourly fluxes represented mean daily fluxes and that daily fluxes changed linearly in between measurements.

### 5. Air, soil moisture and soil temperature

The air temperature and precipitation data were collected from the Hailun State Key Agro-ecological Experiment Station, Hailun County. During gas sampling, soil moisture in the 0–20 cm layer was measured next to the chamber locations. Soil temperatures at 5 cm and 20 cm depth next to the chambers were measured with bent stem thermometers.

### 6. Statistical analysis

The cumulative N_2_O and CO_2_ emissions and average flux were analyzed as a split plot, blocked by year, with season as main effect and fertility treatment as subplot effect. Main plot effects were tested using year*season interaction as error term. Means separation (least significant difference, *P*<0.05) of fertilizer treatments were carried out within each season if the season*fertilizer interaction was significant (*P*<0.05). Additionally, means separation of the yearly winter contribution to total annual N_2_O emissions was carried out. The analyses were conducted using proc glm in SAS (version 9.3, SAS Institute, Cary, NC). Multiple stepwise regression analysis with forward selection of predictor variables using proc reg in SAS was performed to assess the influence of soil temperature, soil moisture, and depth of snow cover on winter N_2_O fluxes.

## Results

### 1. Weather characteristics during the 6-year field measurement

**Figure 1:**
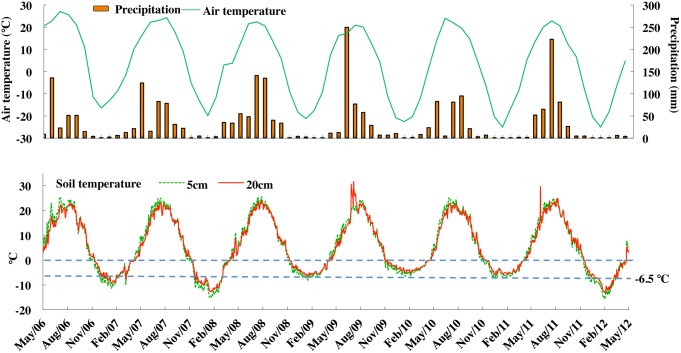
Precipitation, air and soil temperatures. Monthly precipitation and ambient air temperature, and daily soil temperatures at 5 and 20 cm depths from May, 2006 to April, 2012 at Hailun Agroecological Experiment Station, NE China.

In three of the six years, the soil temperature at both 5 cm and 20 cm depths fell below −7°C for at least two months. In the winter 2010/11, such low temperatures lasted only for about three weeks, and in 2008/09 and 2009/10, soil temperatures were −4 to −6°C for about two months. There was approximately 1°C difference in temperature between the two depths (Fig. 1). During the spring/fall season (April & October), mean soil temperatures were on average 4°C and similar between 5 and 20 cm depths. Between 2006 and 2012, >88% of total annual precipitation occurred during the growing season (Fig. 1). In every one of the six years, snowfall occurred only in November and March. In Northeast China, strong winter winds cause thinning of the snowpack during December, January and February. The annual snow amount was less than 20 cm depth though the maximum depth reached up to 40 cm from 2008 to 2010 (S1 Table).

### 2. N_2_O emissions

**Figure 2:**
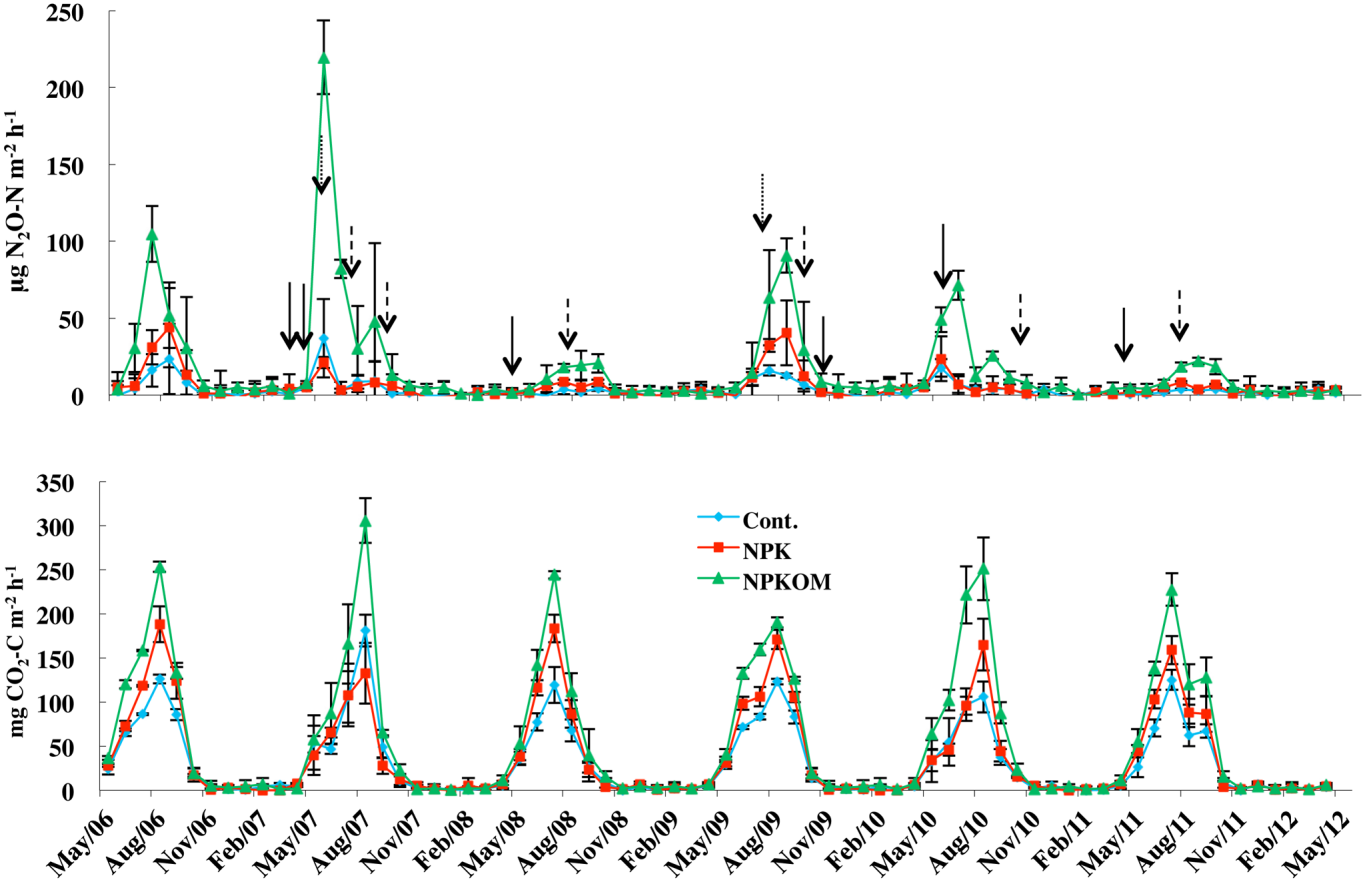
Average hourly N_2_O and CO_2_ fluxes in three fertility management treatments. The monthly average soil-to-atmosphere N_2_O and CO flux in control (Cont. no fertilizer applied), chemically fertilized (NPK) plots, and plots receiving chemical fertilizer and composted pig manure (NPCOM) in the corn-soybean-wheat rotation from May 2006–April 2012. Line bars indicate standard errors (n = 3). Vertical arrows indicate dates of planting (solid lines), harvest (dashed lines) and supplemental fertilizer application (dotted lines).

**Figure 3:**
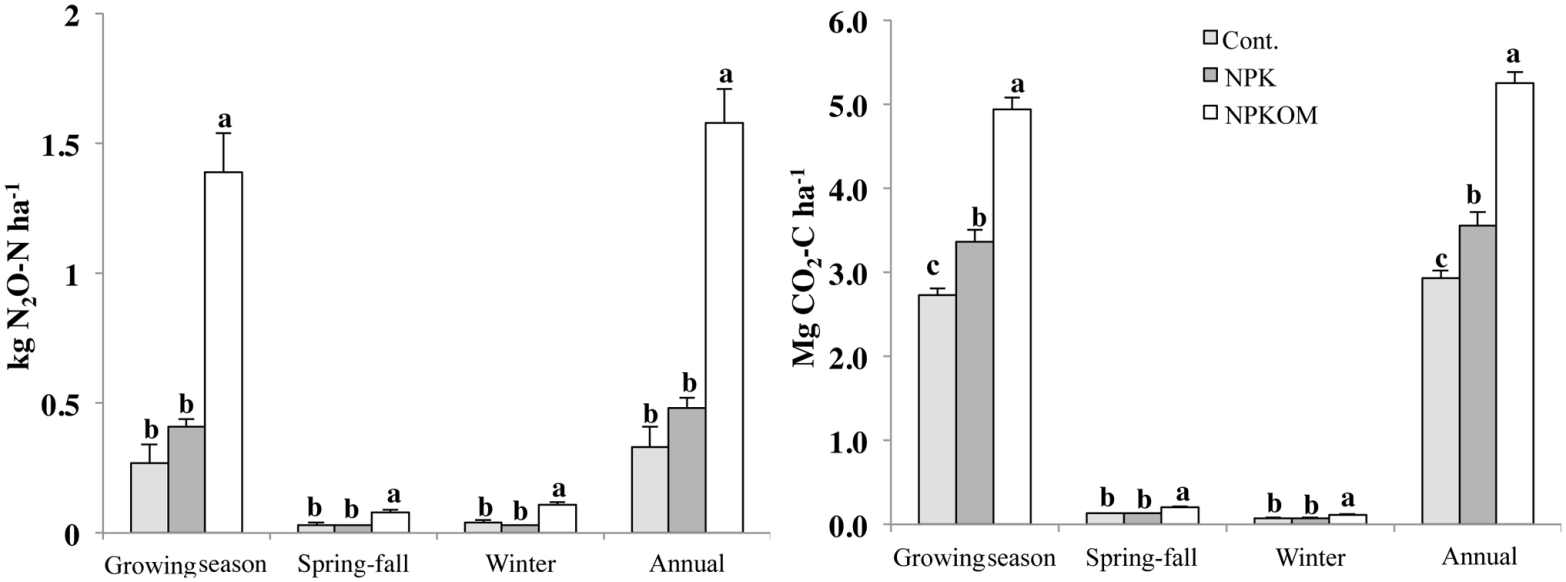
Six-year averages of cumulative seasonal N_2_O and CO_2_ emissions. The treatments were Control (Cont. no fertilizer applied), chemical fertilizer (NPK), and chemical fertilizer plus composted pig manure (NPCOM). Means shown are the average growing season, spring-fall (October and April), winter (November–March), and annual emissions in the corn-soybean-wheat rotation from May 2006–April 2012. Line bars represent standard errors. Bars designated with the same letters within each season are not significantly different (P>0.05). n = 3.

Most of the N_2_O fluxes occurred during the growing season. The magnitude of the peak fluxes varied among the different years although there was no significant overall effect of years on total N_2_O emissions (Fig. 2 and Table 3). The N_2_O emissions were greatest in the NPKOM treatment, while emissions in NPK and Cont. were significantly lower and similar between these two treatments during the growing season, as well as during spring/fall and winter (Fig. 3) (*P<*0.05). The mean soil N_2_O flux in spring/fall ranged from 1.73–5.48 µg N m^−2 ^h^−1^, and from 0.89–3.01 µg N m^−2 ^h^−1^ during winter (Table 4). The estimated N_2_O emissions during the spring/fall seasons were between 0.03 and 0.08 kg N_2_O_–_N ha^−1^ contributing to 5.1–8.8% of the total annual emissions. The contribution of non-growing season to annual N_2_O emissions was 12.5, 12.0, and 21.2% in NPK, NPKOM, and Cont., respectively. In the winter, the N_2_O emissions ranged from 0.03 to 0.11 kg N ha^−1^ and accounted on average for 6.3, 7.0, and 12.1% of annual emissions in NPK, NPKOM and Cont., respectively. The contribution of winter and spring-fall N_2_O emissions to total annual emissions did not differ among years.

**Table 3:** ANOVA results for N_2_O and CO_2_ fluxes and cumulative emissions.

Source ofVariance	N_2_Oflux	CO_2_flux	Cumulative N_2_Oemissions	Cumulative CO_2_emissions
Year	n.s.	n.s.	n.s.	n.s.
Season (S)	***	***	***	***
Fertilizer (F)	***	***	***	***
S×F	**	**	**	**

The average N_2_O and CO_2_ fluxes and cumulative emissions were analyzed as split plot, blocked by year, with season as mainplot effect and fertilizer treatment as subplot effect. n = 3. n.s. = not significant (*P<0.05*).

***P<0.001; **P<0.01. For within season effects, see Table 4 and Fig. 3.

**Table 4:** Mean N_2_O and CO_2_ fluxes by season.

Treatments	Growing season(May–Sep.)	Spring-Fall(Oct. and Apr.)	Winter(Nov.–Mar.)
	µg N_2_O_–_N m^−2 ^h^−1^		
Control	7.24±1.90 b	1.73±0.18 b	1.02±0.24 b
NPK	11.24±0.93 b	2.17±0.31 b	0.89±0.05 b
NPKOM	37.79±4.06 a	5.48±0.56 a	3.01±0.22 a
	**mg CO_2_–C m^−2 ^h^−1^**		
Control	74.32±2.14 c	8.54±0.55 b	2.00±0.08 a
NPK	91.54±4.15 b	9.01±0.36 b	1.86±0.26 a
NPKOM	134.50±3.80 a	13.49±1.75 a	2.95±0.28 a

The average growing season, spring-fall, and winter N_2_O and CO_2_ soil-to-atmosphere fluxes and standard errors under different fertilization treatments. Values designated with the same letter within each season are not different. *P>0.05.* n = 3. NPK synthetic fertilizer applied only; NPKOM synthetic fertilizer and manure applied.

### 3. CO_2_ emissions

The soil CO_2_ flux followed a distinct seasonal pattern in all fertilization treatments (Fig. 2). The highest flux was observed during the summer, with peaks occurring in July and August, and low fluxes were recorded in winter. The CO_2_ fluxes were highest in NPKOM and did not differ between NPK and Cont. (Table 4). Estimated annual soil CO_2_ emissions ranged from 2.93 (Cont.) to 5.25 Mg C ha^−1^ (NPKOM) (Fig. 3). The total winter CO_2_ emissions were 0.07 to 0.11 Mg C ha^−1^, and the total non-growing season soil CO_2_ emission ranged from 0.13 to 0.20 Mg C ha^−1^. The contribution of non-growing season CO_2_ emission to annual soil CO_2_ emission accounted for 5.6 (NPK) to 6.8% (Cont.) of total CO_2_ emissions with winter CO_2_ emissions alone contributing 2.0 (NPK) to 2.4% (Cont.) to annual CO_2_ emissions.

### 4. Relationship between soil N_2_O, CO_2_ flux and soil temperature

**Figure 4:**
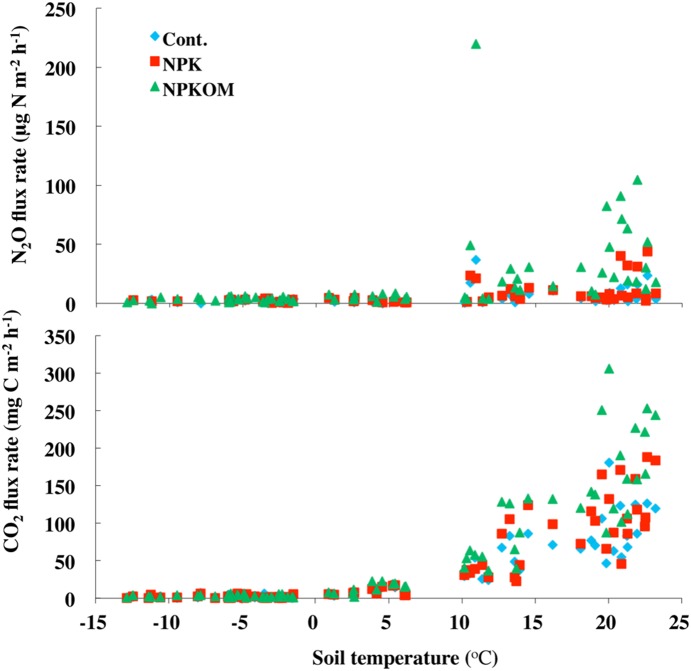
The N_2_O and CO_2_ fluxes in relation to soil temperature at 5 cm depth. The treatments were Control (Cont. no fertilizer applied), chemical fertilizer (NPK), and chemical fertilizer plus composted pig manure (NPKOM), applied in a corn-soybean-wheat rotation from 2006–2012.

There was no relationship between N_2_O flux and temperature although higher fluxes were recorded in summer for the whole growing season (Fig. 4). In contrast, CO_2_ flux increased exponentially with increasing soil temperatures if temperatures were ≥0°C (R^2^>0.82). However, if temperatures <0°C were included in the analysis, there was no significant relationship between CO_2_ flux and soil temperature. The only significant stepwise regression model for winter N_2_O flux (*P*<0.05) was found for the Cont. treatment. The model had a coefficient of determination (r^2^) of 0.10 and included snow cover and soil temperature as predictor variables.

## Discussion

### 1. N_2_O flux during non-growing season

The mean 0.89–3.01 µg N m^−2 ^h^−1^ of N_2_O flux from our continuous winter measurements in this corn-soybean-wheat system is within the range of that reported for agricultural soil in Finland (0.67–23.5 µg N m^−2 ^h^−1^). Both the emission rates and the lowest soil temperature at our site were comparable to those measured by Maljanen et al. [16]. In general, our winter N_2_O flux was lower than most values reported in previous studies (Table 5).

**Table 5:** Comparison of N_2_O fluxes, soil temperatures, and snow depth with other studies.

Location	Year	C(%)	N(%)	MWST at5 cmdepth (°C)	RSD(cm)	Mean flux(µg N m^−2 ^h^−1^)	% of totalannual N_2_Oemissions	Reference
Hailun, China	2006	2.5	0.2	−5.8	0–19	1.8–4.6	7.1–7.4	This study
	2007			−8.3	0–11	0.5–2.1	−0.6–4.3	
	2008			−4.8	0–37	1.3–2.6	12.2–34.5	
	2009			−4.0	0–49	1.4–5.2	3.7–9.5	
	2010			−4.4	0–36	0.6–3.0	−2.0–14.8	
	2011			−7.8	0–7	1.5–2.5	11.9–32.9	
Nebraska, U.S.	1993	1.2	0.1	3.0	NE	2.2	25.0	[34]
Eastern Finland	2004	3.3	NE	0.0	0–430	0.7	NE	[16]
Eastern Finland	2005	1.8	0.2	−6.0	0	57.9	81.0	[13]
South Germany	2008	1.8	NE	0	NE	30.8–113.6	54.2–45.3	[27]
Ontario, Canada	2005	3.1	0.24	−1.0	0–12	12.2–15.0	21.3	[26]
Swiss Alps	2010	8.0	NE	0	0–63	23.2	NE	[38]

Soil carbon and nitrogen, snow depth and ranges, average N_2_O fluxes, and contribution of winter N_2_O emissions to total emissions among studies that reported N_2_O emissions in the cold season in agricultural ecosystems. Abbreviations: C total soil carbon; N total soil nitrogen; NE not estimated; MWST mean winter soil temperature; RSD range of snow depth.

A number of studies showed that deep snowpack promotes moderately cold, stable soil temperatures, which might allow the formation of a cold-adapted microbial community and result in steady winter N_2_O emissions [16], [24]–[27]. However, at our site, the snowpack was relatively thin and decreasing throughout the winter due to wind, and soil temperatures were therefore lower than in those studies. The lack of correlation among winter N_2_O flux and any of the variables snow depth, soil temperature, and soil moisture also supports the conclusion that snow cover did not play a role in enabling N_2_O and CO_2_ production in the surface soil.

Interestingly, we recorded N_2_O fluxes up to 18.84 µg N_2_O_–_N m^−2 ^h^−1^, with average fluxes among the three treatments ranging from 0.33 to 3.68 µg N_2_O_–_N m^−2 ^h^−1^,when soil temperatures were lower than −7.0°C. This temperature was previously considered to be the minimum physiological threshold of soil microbial activity and litter decomposition [15], [28], [29]. To our knowledge, a physiological threshold for N_2_O production has not been established. Under laboratory conditions, psychroactive isolates of microorganisms on ethanol substrate produced CO_2_ and grew exponentially at temperatures as low as −18°C, the only difference in activity from above-zero conditions being severe rate reduction [30]. In our study, N_2_O flux (3.16 µg N_2_O_–_N m^−2 ^h^−1^) was recorded at temperatures as low as −15.41°C (S1 Fig.). The N_2_O emission data seem to suggest a lower threshold for microbial activity than −7°C because the total winter N_2_O emissions were similar in magnitude in the low temperature (years 2007 and 2011) and relatively milder winter (years 2006, 2008–2010) seasons. Thus, adaption of microorganisms to this climate appears to be an explanation for the occurrence of N_2_O emissions at temperatures <−7°C.

An alternative explanation for N_2_O emissions from frozen soil was offered by Maljanen et al. [16] who reported low fluxes of similar magnitude as ours (<3 µg N_2_O_–_N m^−2 ^h^−1^) when temperatures decreased to −15°C at a site without snow cover, but N_2_O concentration in the soil pore space remained at 10 µL L^−1^ until the thawing in spring when soil N_2_O concentration decreased to ambient levels. These researchers suggested that N_2_O was produced during freezing and was related to the increase of microbial available organic carbon from the death of some microbes [16]. At our site in Northeast China, N_2_O may have accumulated in a similar manner and may have been trapped below the frozen layer and then slowly released through the frozen soil during winter.

### 2. CO_2_ flux during non-growing season

The average winter CO_2_ flux rates (1.86–2.95 mg C m^−2 ^h^−1^) from November to March were below 11.36 mg C m^−2 ^h^−1^, as reported in a dry cold season ecosystem with winter mean air temperature from −15 to −5°C [31]. Our data were lower than the mean value of 7.20–13.86 mg C m^−2 ^h^−1^ reported at Dehui Experimental station of Northeast of China [6] and the winter value of 22.46–30.24 mg C m^−2 ^h^−1^ from a corn agro-ecosystem in Northeast China [32]. Soil temperature may have been the main reason for these differences in winter CO_2_ efflux among the different sites (Table 6). In our study, the CO_2_ fluxes showed a similar pattern regardless of small temperature differences among years. The CO_2_ flux did not change with soil temperature variation at the temperatures below 0°C. Similarly, no significant temporal changes in CO_2_ fluxes occurred in a forest-steppe ecotone in north China in winter [33] (Table 6).

**Table 6:** Comparison of CO_2_ fluxes, soil temperatures, and snow depth with other studies.

Location	Year	C(%)	N(%)	MWST at5 cmdepth (°C)	RSD(cm)	Mean flux(mg C m^−2 ^h^−1^)	% of totalannual CO_2_emissions	Reference
Hailun, China	2006	2.5	0.2	−5.8	0–19	0.9–4.1	0.7–2.9	This study
	2007			−8.3	0–11	1.5–2.7	1.1–3.2	
	2008			−4.8	0–37	1.5–3.1	2.2–2.5	
	2009			−4.0	0–49	0.9–4.0	0.8–2.5	
	2010			−4.4	0–36	1.9–2.2	1.4–2.7	
	2011			−7.8	0–7	2.2–2.8	2.0–2.9	
Nebraska, U.S.	1993	1.2	0.1	3.0	NE	1.2	7.0	[34]
Eastern Finland	2008	1.6	0.2	−2.8	0–20	7.3–13.9	5.1–7.1	[6]
Eastern Finland	2004	1.2	0.1	0.9	0–11	12.3–46.4	NE	[32]
South Germany	2006	NE	NE	−5.7	0–30	8.6	5.4	[33]
Ontario, Canada	2010	5.8	0.4	0	0–30	5.0	10.0	[36]
Swiss Alps	2010	8.0	NE	0	0–63	51.0	NE	[38]

Soil carbon and nitrogen, snow depth and ranges, average CO_2_ fluxes, and contribution of winter CO_2_ emissions to total emissions among studies that reported CO_2_ emissions in the cold season in agricultural ecosystems. Abbreviations: C total soil carbon; N total soil nitrogen; NE not estimated; MWST mean winter soil temperature; RSD range of snow depth.

### 3. Contribution of non-growing season soil N_2_O and CO_2_ efflux to annual emission

The contribution of non-growing season N_2_O efflux to annual emission (12.03–21.21%) was comparable to that in other ecosystems, where contributions of 12 to 47% have been reported [25], [34], [35]. Our results show that more than half (50–58%) of the non-growing season N_2_O contribution was due to winter soil N_2_O efflux. The contribution of winter N_2_O emissions to total annual N_2_O emissions did not differ among years although they tended to be higher in 2008 and 2011, which was mainly due to relatively low emissions during the growing season in those two years. The non-growing season soil CO_2_ efflux to annual soil respiration (5.62–6.83%) in the current study was consistent with the results of forest-steppe ecotone (3.48–7.30%) in north of China [33], under different tillage practices in Northeast China (5.1–7.2%) [6], and winter wheat-fallow tillage management system in Sidney, Nebraska (4–10%) [34]. However, our estimated results were lower than CO_2_ emissions from bogs (22%) and fens (10%) in Finland [35], and agricultural land (10%) in Japan [36] (Table 6). In our study, the winter CO_2_ contribution was approximately 1.97 to 2.39% of the annual emission, so the winter CO_2_ emissions contributed much less to non-growing season CO_2_ emissions than winter N_2_O fluxes contributed to non-growing season N_2_O emissions.

### 4. Annual soil N_2_O and CO_2_ emission affected by fertilizer application

Mean annual N_2_O emissions were greatest in the NPKOM treatment, which had 79% greater N_2_O emissions than the control (Fig. 3). In the NPK treatment, N_2_O emissions were 22% greater than in the control. Interestingly, the winter and spring/fall N_2_O and CO_2_ emissions were also greatest in the NPKOM treatment although the average CO_2_ flux in the winter did not differ among the treatments (Table 4). The greater CO_2_ and N_2_O fluxes in the NPKOM treatment was most likely due to the additional available C and N substrates in this treatment [37]. Particularly in the non-growing season, N_2_O emissions from the manure treatment were significantly higher than those of the NPK and Cont. treatments. The manure applications occurred in the fall and likely stimulated N_2_O production. The N_2_O may then have been trapped under the frozen soil after the steep drop in temperature. The fact that in winter both N_2_O and CO_2_ emissions were greater in the treatment receiving organic amendments than in the other treatments supports the conclusion that these gases originated in the surface layer, where the amendments had been applied.

This study showed that non-growing season soil N_2_O and CO_2_ emissions accounted for 12.03–21.21% and 5.62–6.83%, respectively, of the total annual emissions across fertilization treatments in Black soil, northeast China. Thus, the non-growing season, and in particular the winter emissions of N_2_O should be accounted for in estimates of different cropping systems’ annual budgets of N_2_O and CO_2_ loss.

## Supporting Information

S1 Fig **Snow depth, soil moisture and temperature, and N_2_O flux in winter.** Daily values of snow cover, soil volumetric water content in the 0–20 cm layer, soil temperature in the top 5cm layer, and N_2_O flux during the six winter seasons.
(TIF)

S1 Table **Average snow depth, soil moisture and temperature in winter.** Yearly averages of soil volumetric water content, measured next to the chamber bases, in the 0–20 cm layer, soil temperature in the 0–5 cm layer, and snowpack depth during winter seasons.
(DOCX)
