# Supplementary figures and images for: Frozen Cropland Soil in Northeast China as Source of N2O and CO2 Emissions
Source: PLoS One. 2014 Dec 23;9(12):e115761. doi: 10.1371/journal.pone.0115761 (PMC4275265; doi:10.1371/journal.pone.0115761)

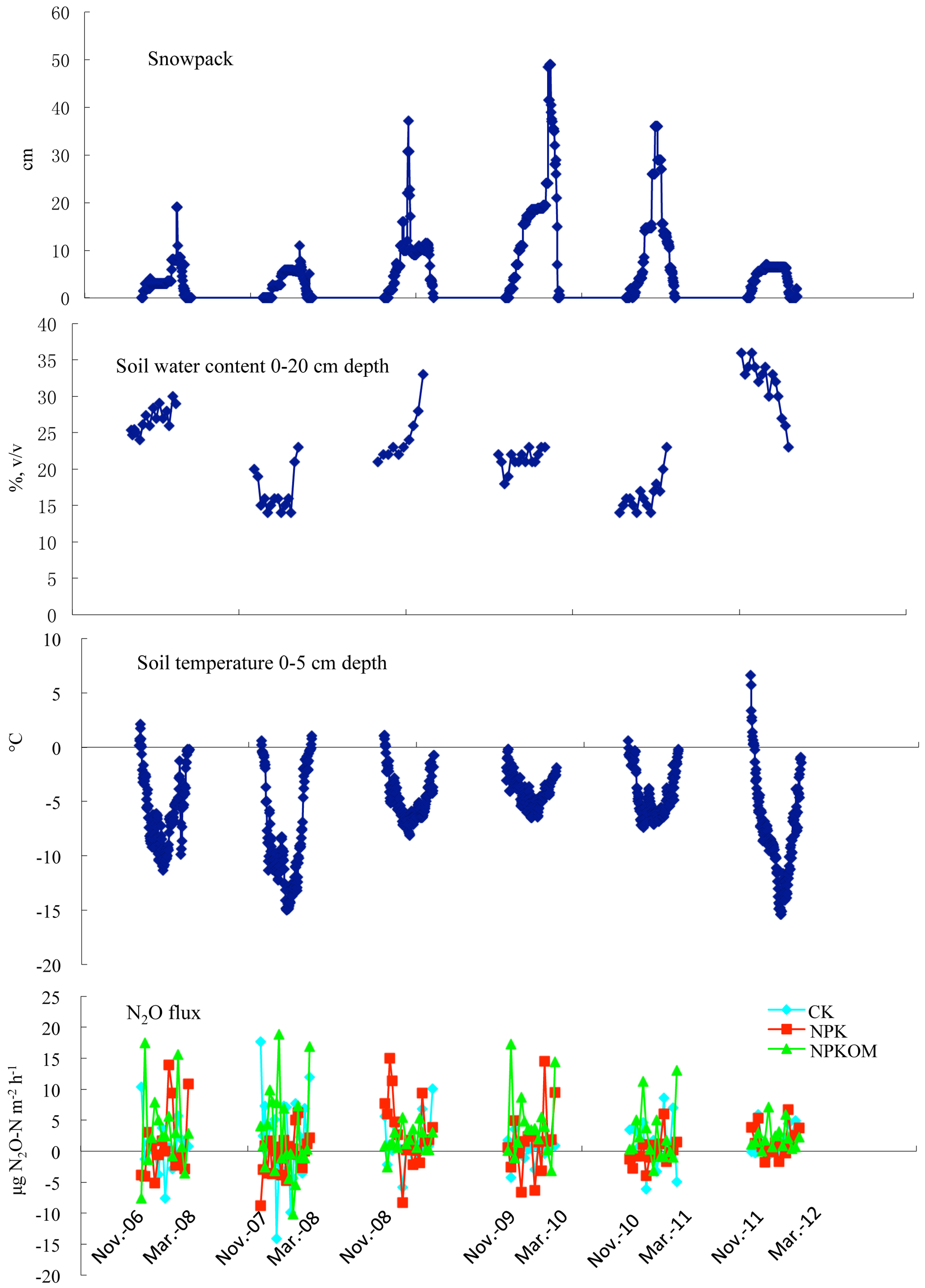

Supplement: S1 Fig — Snow depth, soil moisture and temperature, and N2O flux in winter. Daily values of snow cover, soil volumetric water content in the 0–20 cm layer, soil temperature in the top 5cm layer, and N2O flux during the six winter seasons. (TIF) [file pone.0115761.s001.tif]
