# Supplementary material for: Frozen Cropland Soil in Northeast China as Source of N2O and CO2 Emissions
Source: PLoS One. 2014 Dec 23;9(12):e115761. doi: 10.1371/journal.pone.0115761 (PMC4275265; doi:10.1371/journal.pone.0115761)
Supplement: S1 Table — Average snow depth, soil moisture and temperature in winter. Yearly averages of soil volumetric water content, measured next to the chamber bases, in the 0–20 cm layer, soil temperature in the 0–5 cm layer, and snowpack depth during winter seasons. (DOCX) [file pone.0115761.s002.docx]

**Table S1.** Mean soil moisture, soil temperature, and snowpack depth during winter seasons.

| Year | Soil water content  (v/v, 0-20cm) | Soil temperature at 5 cm depth (^o^C) | Snowpack depth (cm) |
| --- | --- | --- | --- |
| 2006 | 27% | -5.8 | 0-19 |
| 2007 | 17% | -8.3 | 0-11 |
| 2008 | 24% | -4.8 | 0-37 |
| 2009 | 21% | -4.0 | 0-49 |
| 2010 | 16% | -4.4 | 0-36 |
| 2011 | 32% | -7.8 | 0-7 |
